# Local policy governance arrangements and COVID-19-related mortality in municipalities in Japan: a cross-sectional ecological study

**DOI:** 10.3389/fpubh.2025.1622066

**Published:** 2026-01-30

**Authors:** Yuki Nakamoto, Hideki Hashimoto

**Affiliations:** Department of Health and Social Behavior, School of Public Health, The University of Tokyo, Tokyo, Japan

**Keywords:** collaborative governance, COVID-19, governance arrangements, Japan, social determinants of health

## Abstract

**Introduction:**

COVID-19-related mortality varied substantially within and across countries, which may not be fully explained by demographic and clinical differences. Some studies suggest that local governors’ ideology has a substantial effect on mortality because of their political stances for and against the stringency of public health interventions. However, recent research indicates that stringency is not consistently related to lower mortality. This study investigated whether regional differences in COVID-19 mortality are attributable to differences in local governance arrangements for collaborative policy responses to pandemic situations in Japan, where local infection control was managed via a decentralized system.

**Methods:**

From December 2020 to November 2021, we conducted a city-level, cross-sectional, ecological study of 20 cities designated by the government ordinance of Japan. COVID-19-related mortality rates were obtained per 3-month period to account for viral variant change. The local deliberative decision to suspend the national health insurance policy that temporarily revokes the benefit eligibility of households because of unpaid premiums was considered an indicator of flexible and collaborative governance, as the decision required unanimous consensus from diverse stakeholders within and across local government institutes. Negative binomial regression was used adjusting for covariates, including population density and emergency care accessibility.

**Results:**

During the study, 5,383 COVID-19-related deaths were reported. Of the 20 cities, five had implemented the suspension decision before or during the pandemic. Cities with the suspension policy showed an adjusted mortality relative risk of 0.75 (95% confidence interval: 0.60 to 0.94) compared with cities that retained the temporary revocation policy. Conclusions: The results suggest that local governments with flexible collaborative policymaking were more successful in mitigating the COVID-19 mortality impact in Japan. The findings highlight the potentially important effect of governance arrangements on population health disparities.

## 1 Introduction

The concept of social determinants of health is used to explain population health differences across countries, regions, and social strata (1). Previous studies have focused on the social determinants of local government and related public policy administration to explain population-level health outcomes (2–4). The important role of local governments in influencing population health has attracted renewed interest in the USA since the COVID-19 pandemic (5). Several studies indicate that the public policy response in terms of stringent social policies such as mask wearing, mandated vaccination, and lockdown was politicized owing to the political affiliations and ideologies of US governors, resulting in regional variation in COVID-19-related case incidence and mortality (3, 6, 7). However, recent cross-country studies indicate that policy stringency was not consistently related to lower case incidence and mortality; rather, owing to a pattern of reverse causality, higher death rates may have forced governments to adopt more stringent policies (8, 9). One political cross-country analysis also failed to detect an association between policy stringency and lower population mortality, and found that the quality of governance (in addition to population risk factors) was strongly associated with lower mortality (10). Therefore, the aspects of local government that affect population health disparity remain to be determined.

Japan had relatively low case fatality and population mortality during the COVID-19 pandemic (11), although there was a substantial disparity in mortality across regions (12). This disparity may not be fully explained by demographic, socioeconomic, or clinical risk factors, or by medical service accessibility (13). The prefecture with the highest COVID-19-related mortality was Osaka. Its capital, Osaka City, is the second-largest city in Japan and contains a large number of accessible major hospitals. Okinawa Prefecture had relatively moderate COVID-19-related mortality. Okinawa is one of the economically poorest prefectures, and has fewer accessible medical resources owing to its remote island location (14, 15). Japan’s Infection Control Act mandates that local governments are responsible for policy responses to ensure infection control (16). The above-mentioned research suggests that local government responses had an important effect on population outcomes related to the COVID-19 pandemic. It has been argued that coordinated networking within/between levels of government (central, prefecture, and municipality) is essential for effective and timely infection control under Japan’s decentralized governance structure (17).

Previous studies have highlighted the importance of collaboration among multiple stakeholders and local governments in implementing infection control and non-pharmaceutical interventions during the COVID-19 pandemic (18). However, many such studies are case studies and lack empirical analysis of comparisons across municipalities. In this study, we hypothesized that the governance arrangements of local governments were associated with COVID-19-related mortality rates. Specifically, we examined whether the use of government administrative frameworks incorporating flexible policymaking by integrating the perspectives of various sectors and stakeholders was associated with lower COVID-19-related mortality rates.

## 2 Methods

### 2.1 Study design

We used a cross-sectional, city-level, ecological design to test the hypothesis. We used 20 municipality cities designated by the government ordinance of Japan under the Local Government Act as a unit of analysis for two reasons. First, all these cities have populations above 500,000, and an independent local public health center on the frontline of infection control under the governance of the local mayor (19, 20). These cities also have urban economic and health infrastructures. Second, the numbers of COVID-19 cases and deaths per month in the designated cities are publicly available. These cities also manage standard citizen services and have greater autonomy in social welfare, public health, urban planning, management of national roads, and compulsory education (19).

### 2.2 Identification of municipal governments with administrative frameworks for flexible policymaking

Worldwide Governance Indicators are well-known comprehensive measures of country-level governance characteristics (21), although they may not be easily applicable to local governments, which have different powers and responsibilities than central governments. For example, “the kind of voice and accountability” refers to levels of democracy, such as freedom of political participation, expression, and elections, which may not be appropriate to apply at the local government level (22).

Because of the lack of standardized and applicable measures for our purpose, we chose a local policy of temporary benefit revocation for unpaid premiums under the national health insurance system to measure the extent to which local governments used administrative frameworks to manage policy consensus from various sectors and stakeholders. The rationale for this is as follows.

Japan’s public national health insurance system provides universal cover for community residents and is implemented by local government insurers under the National Health Insurance Act (23). The system mandates premium contributions according to household size and income level, and offers full medical, dental, and pharmaceutical coverage with a standardized copayment rate. However, Article 9 of the Act stipulates that individuals who default on their premium payments for a year or longer will be temporarily ineligible for coverage when utilizing healthcare services, and will be subject to full out-of-pocket medical visit expenses until the premium payments are no longer in arrears (24). During the period of ineligibility, the local municipality’s government insurers are mandated to issue a “certification of beneficiary status,” which guarantees that out-of-pocket payments will be reimbursed after confirmation of premium payments.

Although the issuance of status certification is generally mandated, an exemption may be applied if the local government makes a policy decision to suspend the temporary revocation of benefits. Such decisions are approved following unanimous consensus across the mayor’s office, city council, and other related parties, and are made for various reasons, including welfare (e.g., to ensure equal benefits for poorer households) or for management (e.g., avoiding the costs involved in temporarily revoking benefits).

Regardless of the reason for these decisions, implementing and maintaining the suspension policy necessitates complex policy processes that require coordination and unanimous consensus between the mayor, the insurance bureau, related sectors in the municipality office, the municipal council, and other internal and external actors whose interests and political values vary widely. Following the National Health Insurance Act, Local Councils for the Administration of National Health Insurance (statutory advisory councils), which comprise representative insured persons and medical providers, are required to submit an advisory opinion to the mayor on whether the suspension policy is relevant and feasible in ensuring equality of both benefits and contributions (24). The city councils have the power to determine the legitimacy of the suspension policy in relation to the city budget (25), and the prefecture functions as a supervisory agency for municipality insurer offices for jurisdiction and bureaucratic feasibility (24). These complex processes mean that local governments that intend to adopt the suspension policy must have the administrative capacity to align divergent perspectives across sectors and stakeholders. The required governance framework corresponds to the “collaborative governance” model, which involves collaboration across diverse stakeholders in multiple sectors to address public service issues through collective efforts, promoting flexible actions based on trust and legitimacy (26).

**Figure 1:**
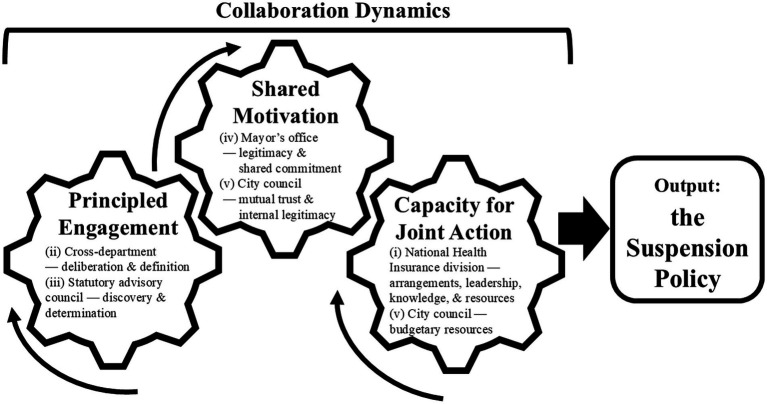
Collaboration dynamics as the necessary condition for the suspension policy. Principled engagement involves discovery, definition, deliberation, and determination. Shared motivation refers to mutual trust, mutual understanding, internal legitimacy, and shared commitment. Capacity for joint action comprises procedural and institutional arrangements, leadership, knowledge, and resources. Curved arrows denote iterative interactions; the rightward arrow denotes translation into the suspension policy. Labels (i–v) indicate the primary association between approval/coordination domains and each element; associations are non-exclusive, and no unique order is implied.

The suspension policy is implemented through processes involving multiple stakeholders and iterative activities across five interrelated functions (Figure 1): (i) groundwork laid by the National Health Insurance division; (ii) coordination between the legal, finance, and revenue-collection departments; (iii) deliberation by the statutory advisory council; (iv) decision-making by the mayor’s office; and (v) authorization and budgeting by the city council. These functions interact and repeat rather than unfolding as a fixed sequence of discrete tasks, and they require cooperation across both administrative and political entities.

In alignment with Emerson et al. (26) collaboration dynamics framework, this process unfolds through iterative interactions between three domains: principled engagement, which structures discovery, definition, deliberation, and determination; shared motivation, which involves building mutual trust, mutual understanding, internal legitimacy, and shared commitment; and capacity for joint action, characterized by concrete procedural and institutional arrangements, leadership, knowledge, and resources. We therefore consider the adoption of the suspension policy as a collaborative governance outcome that arises from iterative, mutually reinforcing interactions between principled engagement, shared motivation, and capacity for joint action throughout this governance process. Figure 1 illustrates how these functions relate to the three domains. Thus, we interpret this publicly visible decision as a stringent test of municipal collaborative governance capacity.

The implementation of such complex policymaking processes, particularly the suspension policy, is unlikely to simply reflect an ideological motivation to attract voting interests from liberal parties. This is because only 0.2% of households in Japan are subject to temporary benefit revocation (27, 28), and there is strong support for benefit revocation as a way of ensuring contribution fairness. Thus, contrary to the US discussion about the politicization of pandemic control, the suspension policy cannot be fully explained by the dominant ideology of local government.

Additionally, the suspension policy per se did not theoretically affect accessibility to medical treatment for COVID-19, as the Central Government Ministry announced conditional exceptions to the revocation policy for COVID-19-related consultations as of November 2020 (29), before the sampling period of the current study began. The aim of these exceptions was to facilitate equitable access to required medical services for people in poverty. Although access to medical care for non-COVID-19 conditions was discouraged owing to the revocation of benefit eligibility (30), this would not have affected the COVID-19-related mortality rate. Thus, any association between the policy of suspending benefit eligibility revocation and reductions in COVID-19-related mortality in a municipality should be related to municipal-level management of pandemic-related policy decisions (17, 31) rather than subsequent improvement of medical care access for people in poverty. We argue that the suspension policy may be an indicator of the governance style of local governments that used coordinated and collaborative policy management across health and non-health sectors to effectively respond to the ongoing challenges of the pandemic.

We treat policy outputs as observable manifestations of collaborative governance. Among potential outputs, suspension was selected as the indicator because it is formally decided and documented, requires non-sequential alignment across the domains described above, and is conceptually anchored in the three collaboration dynamic domains operating iteratively (Figure 1). This theory-guided operationalization provides content validity for municipal-level inference.

### 2.3 Data source

The monthly cumulative number of confirmed COVID-19-related deaths was the study outcome (32). Death data from December 2020 to November 2021 were compiled quarterly to reflect different COVID-19 variant outbreaks. Before December 2020, the original strain dominated the first period, and the Alpha variant dominated the second period in Japan. During the study period, the Delta variant became more prevalent; this was followed by the Omicron variant outbreak, during which the death count rapidly increased (16).

To measure city-level governance arrangements, we collected data on the suspension or continuation of temporary benefit revocation in each city by reviewing the minutes of meetings of its city council and the Local Council for the Administration of National Health Insurance. In July 2023, we confirmed that information by phone calls to each city. Cities with a policy of suspending temporary benefit revocation under the national health insurance system were assumed to have governments with administrative frameworks for flexible and collaborative policymaking (as argued above). Because significant changes in governance typically require more than 5 years (33), we classified cases as having a suspension policy from the beginning of the observation period, even if the policy was instituted during that time.

The following city-level covariates were measured: (1) proportion of the population aged 75 years or over (27), (2) number of acute care hospital beds per population (27, 34), (3) proportion of nursing home residents (27, 35), and (4) population density (in thousands of people per km^2^) (27, 36), as factors that may affect death rates, following a previous study (13). The specific covariate formulas are shown in Table 1.

**Table 1:** Data sources and calculation methods for covariates.

Covariate	Calculation method
(1) Proportion of population aged ≥75 years	Population aged ≥75 years (27)/City population (27)
(2) Number of acute care hospital beds per population	Number of beds in emergency hospitals (34)/City population (27)
(3) Proportion of nursing home residents	(Number of persons using nursing homes over 12 months (35)/12 − Residents in facilities outside the city (35))/City population (27)
(4) Population density	City population (27)/1,000/Square kilometers of habitable land area (36)

### 2.4 Statistical analysis

We used negative binomial regression to address over-dispersion (37). This method was applied to assess the association between COVID-19-related deaths and the suspension of temporary benefit revocation, adjusting for covariates and periods across the 20 cities. We designated the first 3 months as the reference period. The logarithmic value of the number of deaths at the city level was set as the outcome variable. To account for differences in city population size, we included the logarithmic value of the city population as an offset term. Additionally, we clustered standard errors at the city level to account for within-city correlations across periods. All analyses were conducted using Stata/IC version 14.2 (Stata Corporation, College Station, Texas, USA).

We adhered to the Strengthening the Reporting of Observational Studies in Epidemiology (STROBE) reporting guideline (38). Institutional review board approval was waived for this study, which did not require informed consent, as it used deidentified, publicly available data.

## 3 Results

From December 2020 to November 2021, 5,383 confirmed COVID-19 deaths were reported in the 20 designated cities. Five cities had implemented a policy to suspend the temporary revocation of eligibility for benefits; at the time of our phone confirmation, the other cities had not implemented this policy. Yokohama City implemented this policy before 2020, three cities implemented it at the beginning of 2020, and another city implemented it in August 2021. The suspension policy was implemented in four cities to equalize benefits for households in poverty and in another city to avoid management concerns (e.g., avoiding the costs involved in temporarily revoking benefits). The implementation of the suspension measure was documented in publicly released council or committee minutes for all five adopting cities, consistent with our telephone confirmations (Supplementary Table 1).

Between December 2020 and February 2021, the death rate per 1,000,000 people was 50.52 in cities that had implemented the suspension policy and 62.68 in cities that had not (Table 2). This trend persisted in the following periods, with lower mortality rates in cities with the suspension: 30.68 versus 89.03 in the second period and 30.68 versus 44.49 in the third period. However, this trend changed in the fourth period: cities that had implemented the suspension policy had a rate of 24.68, compared with 23.32 in those that had not. We observed significantly reduced mortality in the fourth period, when vaccination coverage reached 75% of the population and plateaued (39), and the difference in mortality rates between governance arrangements was lower (Table 2).

**Table 2:** Comparison of city demographic and health infrastructure characteristics and COVID-19 mortality rates in four periods.

Characteristic	Suspension of temporary benefit revocation (*n* = 5)		Continuation of temporary benefit revocation (*n* = 15)	
	Mean	SD	Mean	SD
Population*	1,733,985	1,318,030	1,275,275	565,357
COVID-19 mortality in four periods (per million)				
Dec 2020–Feb 2021	50.52	22.31	62.68	31.86
Mar 2021–May 2021	30.68	8.47	89.03	85.56
Jun 2021–Aug 2021	30.68	9.48	44.49	29.00
Sep 2021–Nov 2021	24.68	12.08	23.32	11.66
Proportion of population aged ≥75 years (%)	12.72	0.46	13.18	1.71
Number of acute care hospital beds per population (%)	0.60	0.21	0.69	0.17
Proportion of nursing home residents (%)	0.53	0.02	0.56	0.17
Population density (1,000 people / km^2^)	4.75	3.14	4.38	2.90

Weighted means and standard deviations (SD) are shown; the population variable was excluded.

*Population ranges.

- Suspension of temporary benefit revocation: 3,777,491–724,691.

- Continuation of temporary benefit revocation: 2,752,412–693,389.

The results of the negative binomial regression model are shown in Table 3. Cities with the suspension policy had a relative mortality rate of 0.75 (95% confidence interval: 0.60–0.94) compared with their counterpart cities that continued the revocation policy. The periodical relative mortality risks (compared with the first period) were 1.11 (0.78–1.58) in the second period, 0.71 (0.60–0.85) in the third period, and 0.44 (0.34–0.58) in the fourth period. Additionally, having a greater proportion of the population aged ≥75 years was related to a higher relative risk of COVID-19 mortality of 1.37 (1.17–1.60). A higher number of acute care hospital beds per capita was associated with a relative risk of 0.98 (0.54–1.78). A higher proportion of nursing home residents per capita was associated with a lower relative risk of 0.05 (0.01–0.28). Finally, greater population density was associated with a higher relative risk of 1.12 (1.08–1.17).

**Table 3:** Results of negative binomial regression for COVID-19 mortality in four periods (*n* = 80).

Variable	Relative risk (95% CI)	
Suspension of temporary benefit revocation	0.75	(0.60–0.94)
In four periods		
Dec 2020–Feb 2021	Ref	
Mar 2021–May 2021	1.11	(0.78–1.58)
Jun 2021–Aug 2021	0.71	(0.60–0.85)
Sep 2021–Nov 2021	0.44	(0.34–0.58)
Proportion of population aged ≥75 years (%)	1.37	(1.17–1.60)
Number of acute care hospital beds per population (%)	0.98	(0.54–1.78)
Proportion of nursing home residents (%)	0.05	(0.01–0.28)
Population density (1,000 people / km^2^)	1.12	(1.08–1.17)

Mortality rate ratios were calculated using log (population) as the offset.

Robust standard errors clustered at the city level.

CI, confidence interval.

We assessed robustness using equivalized disposable household income, Poisson regression, exclusion of the two largest cities, and diagnostics for multicollinearity. All sensitivity checks yielded essentially similar inferences (Supplementary Tables 2–5).

## 4 Discussion

The current study identified an association between municipality adoption of the suspension of temporary benefit eligibility revocation under the national health insurance system and lower COVID-19 mortality, adjusting for period, demographics, healthcare resources, and population density. The results suggest that cities with administrative governance frameworks for coordinated policymaking demonstrated better management of COVID-19 mortality compared with their counterpart cities. The findings align with the results of previous national-level studies, and indicate that governance may affect population health outcomes (10).

The observed mortality differences were unlikely to be attributable to the suspension policy per se for several reasons. First, only approximately 0.2% of the total households in Japan are subject to temporary benefit revocation (27, 28); this percentage is too small to explain the magnitude of the estimated risk ratio. Additionally, medical visits related to COVID-19 were subsidized for households in poverty during the study period (29); thus, it is unlikely that poorer households subject to the temporary revocation had very high case fatalities.

The political ideology of state chief executives may lead to different stringent policy choices, as observed in the USA (6). In our sample cities, five mayors who implemented the policy ran for the post of mayor without any party affiliation, and four mayors were considered to be conservatives (40–45). Thus, it is not necessarily the case that the suspension policy decision was based on an ideology of liberalism/welfarism. Moreover, governance arrangements, regardless of the political ideology of the leader, often require balanced management of the various values and interests of stakeholders (46, 47).

Regarding the covariates included in the model, a higher proportion of nursing home residents per capita was associated with lower municipal mortality. This association is counterintuitive, but is consistent with the prioritization of early vaccination for long-term care residents and staff in Japan, along with facility-level controls (such as restricted visits and outings) that disproportionately protected this group (48, 49). Given the ecological design, we interpret this as a population-level association rather than individual protection. Higher mortality rates were observed in those regions that contained more people aged ≥75 years and those with denser populations, which is consistent with previous findings (50, 51).

The COVID-19 vaccination program in Japan was rolled out in phases, beginning with healthcare workers in February 2021 and expanding to older adults and other high-risk groups from April 2021 (48). Our data showed that between-group mortality differences narrowed in the fourth period, indicating that any association with the suspension policy arose primarily pre-vaccination. This pattern is consistent with US evidence that vaccination attenuated partisan differences in excess mortality (52) and suggests that collaborative governance—for case tracing and triage—mattered most in the early pandemic phase when there was considerable uncertainty (31).

Governance arrangements affect the formation of healthcare systems (53). The results of the current study suggest several pathways by which local governance arrangements resulted in population health outcomes during the pandemic. During the pandemic period, local public health centers were the focus of coordination with healthcare facilities and other entities for managing hospitalizations, health monitoring, and contact tracing of COVID-19 patients during case surges. However, many centers were stretched beyond capacity by the rapid increase in cases (16). Healthcare facilities also were challenged by the limited number of beds and medical staff to care for COVID-19 inpatients, and had to reallocate their treatment provision from patients with other diseases to those with COVID-19 (54). Some local governments addressed these difficulties by sharing data on hospitalized COVID-19 patients and available beds using a unified assessment scale for hospital admissions in coordination with healthcare providers and public health centers, as well as using information and communications technology to monitor mild cases (54). These flexible measures required reallocating staff from various departments and collaborating with healthcare institutions, health and non-health sectors of local governments, and private hospitals. Our results indicate that successful governance arrangements involved efficient resource allocation and information sharing to effectively manage the surge in cases.

Similar challenges in organizing local and regional responses to COVID-19 have been reported outside Japan (3, 5–7, 18, 55–61). These examples also highlight the importance of coordinating health and social policies across sectors when responding to population health challenges during the pandemic. The current study, although limited to analysis of city-level data from Japan, suggests that similar mechanisms might operate at national and international levels. However, it remains unclear who benefits most from governance differences. To enable more systematic comparisons across contexts, future work should refine and extend indicators of multifaceted governance; for example, by constructing comparable measures based on publicly visible policy decisions in other countries. Such indicators could be used to examine how collaborative governance shapes key social determinants of health and contributes to widening or narrowing socioeconomic disparities in mortality and access to care.

### 4.1 Limitations

This study had several limitations. First, the design was cross-sectional and ecological at the municipal level; therefore, causal relationships cannot be inferred and the findings are not interpretable at the individual level. Given the limited sample size and the likelihood of residual or unmeasured confounding, as well as potential policy endogeneity and reverse causation, the observed patterns should be viewed as associations and as indicators that help to generate future hypotheses. Second, categorizing governance arrangements according to a single policy may have led to problems with misclassification. Although other municipal-level indicators of collaborative governance may exist, in this setting we considered the suspension policy a practice-grounded proxy for municipal collaborative governance; broader indicator sets should be developed in future studies. Four cities implemented the policy before the observation period, and one city during this period. Additionally, 2 of the 20 designated cities changed mayors during the observation period (42). However, substantial changes in governance arrangements may require an extended period, such as ≥5 years, and we believe there were no good reasons for major governance style changes to occur during the observed 2-year window (33). Third, although socioeconomic status was not included in the primary adjustment set, we conducted a sensitivity analysis by adding a city-level socioeconomic status proxy—equivalized disposable household income per capita—and the results were essentially unchanged (Supplementary Table 2). A previous study identified associations between socioeconomic status and COVID-19-related mortality and incidence at the prefecture level in Japan; in particular, a higher unemployment rate was proportionally linked to higher mortality (13). However, the differences in unemployment rates across the 20 cities were less pronounced than the differences across the prefectures (27). Therefore, although the sensitivity analysis reduces concerns about confounding among socioeconomic status variables, the presence of some residual confounding cannot be ruled out. Fourth, this study included only 20 ordinance-designated cities, which typically have greater administrative and public health capacities than smaller municipalities. It may not be possible to generalize the findings to smaller Japanese municipalities, prefectural public health center jurisdictions, and international settings; the coordination mechanisms we propose may be transferable, but effect sizes are likely to depend on local capacity, provider market structure, and legal authority. Finally, owing to the lack of disaggregated data, we compared crude rates of COVID-19-related mortality, which may have ignored important variations in sex and age distributions among city populations.

## 5 Conclusion

In this study, we identified a correlation between the policy of suspending temporary benefits under the national health insurance system of Japan and reduced COVID-19 mortality at the city level. Our findings indicate the potential effectiveness of governance arrangements in providing public health services. Further research is recommended to explore the mediating pathways by which governance arrangements affect public health, and to develop governance arrangement indicators.

## Data Availability

All the data used in this study are publicly available from the cited databases. The telephone survey results are included in the manuscript and Supplementary Table 1.
