# Supplementary material for: Local policy governance arrangements and COVID-19-related mortality in municipalities in Japan: a cross-sectional ecological study
Source: Front Public Health. 2026 Jan 30;13:1622066. doi: 10.3389/fpubh.2025.1622066 (PMC12901323; doi:10.3389/fpubh.2025.1622066)
Supplement: Supplementary file 1 [file Table_1.docx]

**Supplementary Table 1**. Sources and verification used to code the city-level suspension of the temporary National Health Insurance benefit revocation.

| City | Adoption date | Stated rationale | Public documentary sources |
| --- | --- | --- | --- |
| Sendai | Jun 2020- | Equalize benefits for households in poverty | City council minutes (1) |
| Yokohama | Oct 2016- | Avoid management concerns | City council minutes (2) |
| Nagoya | March 2020- | Equalize benefits for households in poverty | City council minutes (3) |
| Okayama | August 2021- | Equalize benefits for households in poverty | City council minutes (4) |
| Kumamoto | March 2020- | Equalize benefits for households in poverty | City council minutes (5) |

Supplementary References

1. Sendai City Council, Japan (n.d.). Sendai City Council Reiwa 4th Year 2nd Regular Meeting *(in Japanese)*. https://www.city.sendai.miyagi.dbsr.jp/index.php/4931216?Template=view&VoiceType=all&DocumentID=3290  [Accessed October 23, 2025].

2. Yokohama City Council, Japan (n.d.). Yokohama City Council Reiwa First Fiscal Year Financial Statements First Special Committee October 2, 2020 (in Japanese). http://giji.city.yokohama.lg.jp/tenant/yokohama/MinuteView.html?council_id=788&schedule_id=10&is_search=false&view_years=2020 [Accessed October 23, 2025].

3. Nagoya City Council (n.d.). Nagoya City Council, Reiwa 2nd Year Finance and Welfare Committee, March 10, 2020(in Japanese). https://ssp.kaigiroku.net/tenant/nagoya/SpMinuteView.html?power_user=false&tenant_id=207&council_id=530&schedule_id=8&view_years=2020 [Accessed October 23, 2025].

4. Okayama City Council, Japan (n.d.). Okayama City Council Reiwa 3rd Year August Regular Meeting, September 6, 2021 (in Japanese).

https://ssp.kaigiroku.net/tenant/okayama/MinuteView.html?council_id=435&schedule_id=3&is_search=true [Accessed October 23, 2025].

5. Kumamoto City Council, Japan (n.d.). Kumamoto City Council Reiwa 2nd Year 1st Regular Meeting, March 24, 2020 (in Japanese). http://kumamoto.gijiroku.com/voices/g08v_viewh.asp?Sflg=11&FYY=2020&TYY=2020 [Accessed October 23, 2025].
