# Supplementary material for: Local policy governance arrangements and COVID-19-related mortality in municipalities in Japan: a cross-sectional ecological study
Source: Front Public Health. 2026 Jan 30;13:1622066. doi: 10.3389/fpubh.2025.1622066 (PMC12901323; doi:10.3389/fpubh.2025.1622066)
Supplement: Supplementary file 2 [file Table_2.docx]

**Supplement Table 2**. Results of negative binomial regression for COVID-19 mortality in four periods with equivalized disposable household income per capita (n=80).

|  | Relative risk (95% CI) | | | | | |
| --- | --- | --- | --- | --- | --- | --- |
| Suspension of temporary benefit revocation | 0.74 | ( | 0.59 | – | 0.93 | ) |
| in four periods |  |  |  |  |  |  |
| Dec 2020–Feb 2021 | ref |  |  |  |  |  |
| Mar 2021–May 2021 | 1.10 | ( | 0.77 | – | 1.57 | ) |
| Jun 2021–Aug 2021 | 0.71 | ( | 0.60 | – | 0.85 | ) |
| Sep 2021–Nov 2021 | 0.44 | ( | 0.34 | – | 0.58 | ) |
| Proportion of population aged ≥75 years (%) | 1.37 | ( | 1.17 | – | 1.60 | ) |
| Number of acute care hospital beds per population (%) | 0.95 | ( | 0.47 | – | 1.94 | ) |
| Proportion of nursing home residents (%) | 0.05 | ( | 0.01 | – | 0.27 | ) |
| Population density (1,000 people / km^2^) | 1.12 | ( | 1.08 | – | 1.17 | ) |
| Equivalized disposable household income per capita (per 100,000 JPY) | 0.94 | ( | 0.52 | – | 1.72 | ) |
| Mortality rate ratios were calculated using log (population) as the offset. | | | | | | |
| Robust standard errors clustered at the city level. | | | | | | |
| Using equivalized disposable household income per capita from the Family Income and Expenditure Survey, 2020 (1) | | | | | | |

Supplementary References

(1) Statistics Bureau of Japan. Family Income and Expenditure Survey(n.d.). https://www.stat.go.jp/english/data/kakei/index.html [Accessed October 23, 2025]
