# Supplementary material for: Local policy governance arrangements and COVID-19-related mortality in municipalities in Japan: a cross-sectional ecological study
Source: Front Public Health. 2026 Jan 30;13:1622066. doi: 10.3389/fpubh.2025.1622066 (PMC12901323; doi:10.3389/fpubh.2025.1622066)
Supplement: Supplementary file 3 [file Table_3.docx]

**Supplementary Table 3**. Results of Poisson regression for COVID-19 mortality in four periods (n=80).

|  | Relative risk (95% CI) | | | | | |
| --- | --- | --- | --- | --- | --- | --- |
| Suspension of temporary benefit revocation | 0.69 | ( | 0.51 | – | 0.94 | ) |
| in four periods |  |  |  |  |  |  |
| Dec 2020–Feb 2021 | ref |  |  |  |  |  |
| Mar 2021–May 2021 | 1.20 | ( | 0.81 | – | 1.79 | ) |
| Jun 2021–Aug 2021 | 0.68 | ( | 0.57 | – | 0.82 | ) |
| Sep 2021–Nov 2021 | 0.40 | ( | 0.31 | – | 0.53 | ) |
| Proportion of population aged ≥75 years (%) | 1.40 | ( | 1.18 | – | 1.68 | ) |
| Number of acute care hospital beds per population (%) | 1.24 | ( | 0.57 | – | 2.66 | ) |
| Proportion of nursing home residents (%) | 0.06 | ( | 0.01 | – | 0.46 | ) |
| Population density (1,000 people / km^2^) | 1.12 | ( | 1.07 | – | 1.18 | ) |
| Mortality rate ratios were calculated using log (population) as the offset. | | | | | | |
| Robust standard errors clustered at the city level. | | | | | | |
