# Supplementary material for: Local policy governance arrangements and COVID-19-related mortality in municipalities in Japan: a cross-sectional ecological study
Source: Front Public Health. 2026 Jan 30;13:1622066. doi: 10.3389/fpubh.2025.1622066 (PMC12901323; doi:10.3389/fpubh.2025.1622066)
Supplement: Supplementary file 4 [file Table_4.docx]

**Supplementary Table 4**. Results of negative binomial regression for COVID-19 mortality in four periods without the two largest cities (n=72).

|  | Relative risk (95% CI) | | | | | |
| --- | --- | --- | --- | --- | --- | --- |
| Suspension of temporary benefit revocation | 0.74 | ( | 0.52 | – | 1.07 | ) |
| in four periods |  |  |  |  |  |  |
| Dec 2020–Feb 2021 | ref |  |  |  |  |  |
| Mar 2021–May 2021 | 1.09 | ( | 0.74 | – | 1.59 | ) |
| Jun 2021–Aug 2021 | 0.72 | ( | 0.59 | – | 0.87 | ) |
| Sep 2021–Nov 2021 | 0.43 | ( | 0.32 | – | 0.58 | ) |
| Proportion of population aged ≥75 years (%) | 1.36 | ( | 1.16 | – | 1.60 | ) |
| Number of acute care hospital beds per population (%) | 0.78 | ( | 0.38 | – | 1.61 | ) |
| Proportion of nursing home residents (%) | 0.04 | ( | 0.01 | – | 0.21 | ) |
| Population density (1,000 people / km^2^) | 1.08 | ( | 1.00 | – | 1.16 | ) |
| Mortality rate ratios were calculated using log (population) as the offset. | | | | | | |
| Robust standard errors clustered at the city level. | | | | | | |
| Excludes Yokohama and Osaka—Japan’s two most populous ordinance-designated cities. | | | | | | |
