# Supplementary material for: Local policy governance arrangements and COVID-19-related mortality in municipalities in Japan: a cross-sectional ecological study
Source: Front Public Health. 2026 Jan 30;13:1622066. doi: 10.3389/fpubh.2025.1622066 (PMC12901323; doi:10.3389/fpubh.2025.1622066)
Supplement: Supplementary file 5 [file Table_5.docx]

**Supplementary Table 5**. Variance inflation factors (VIF) for covariates in the main model.

|  | VIF |
| --- | --- |
| Suspension of temporary benefit revocation | 1.16 |
| in four periods |  |
| Dec 2020–Feb 2021 | ref |
| Mar 2021–May 2021 | 1.50 |
| Jun 2021–Aug 2021 | 1.50 |
| Sep 2021–Nov 2021 | 1.50 |
| Proportion of population aged ≥75 years (%) | 2.51 |
| Number of acute care hospital beds per population (%) | 1.29 |
| Proportion of nursing home residents (%) | 2.31 |
| Population density (1,000 people / km^2^) | 1.66 |
| Mean VIF | 1.68 |
